# Influenza A(H1N2)v: global impact, emerging threats and preventive measures

**DOI:** 10.1097/MS9.0000000000001948

**Published:** 2024-03-12

**Authors:** Emmanuel Kokori, Gbolahan Olatunji, Ayomikun Mokuolu, Aminat Akinoso, Ibukun Olunlade, Innocent Shu Bonu, Badrudeen Olalekan Alabi, Joy Chidinma Oguaju, Nicholas Aderinto

**Affiliations:** a Department of Medicine and Surgery, University of Ilorin, Ilorin; b Oba Ademola Maternity Hospital Ijemo, Abeokuta; c Department of Medicine, Ladoke Akintola University of Technology, Ogbomoso, Nigeria; d Emergency Department, Regional Hospital Bamenda, Bamenda, Cameroon

## Introduction

Influenza, often called the flu, is a highly contagious respiratory illness caused by influenza viruses, targeting the respiratory tract encompassing the nose, throat, and lungs, as reported by the CDC^1^. While the well-documented and closely monitored influenza types A and B are prevalent, a novel variant called Influenza A(H1N2)v, also known as Swine Influenza, was officially reported to the Pan American Health Organization (PAHO)/WHO by the United States IHR National Focal Point on August 4, 2023^2^. This marks the 37th human infection with the Influenza A(H1N2)v virus in the United States since 2005^2^. These swine-origin Influenza A(H1N2) viruses are known to circulate among swine populations worldwide. Human infections typically occur through direct or indirect exposure to pigs or their contaminated environments, leading to symptoms like fever, fatigue, reduced appetite, coughing, and occasionally, a runny nose, sore throat, eye irritation, nausea, vomiting, and diarrhoea^1^.

## The case and response

The case involved an individual under 18, a Michigan, USA, resident with no underlying health conditions. On 29 July 2023, the patient developed respiratory illness, displaying symptoms including fever, cough, sore throat, muscle aches, headache, shortness of breath, diarrhoea, nausea, dizziness, and lethargy. Seeking medical care at an emergency department on 29 July an upper respiratory tract specimen collected on July 30th tested positive for influenza A virus on the same day. Fortunately, hospitalisation was not necessary.

In response to the case, local public health officials launched an investigation. They identified swine exposure during an agricultural fair attended by the patient between 23 and 29 July, 10 days before illness onset. Further investigation did not uncover respiratory illness among the patient’s close contacts or household members^2^. Following the incident, local health officials initiated active surveillance for cases. Response efforts included active case finding among fair exhibitors and their families by the county health department and notifying local providers to monitor individuals who had attended the fair or had recent swine contact for respiratory illness^2^.

## Understanding Influenza A(H1N2)v

Influenza viruses are categorised into four types: A, B, C, and D. Seasonal flu epidemics in humans are mainly caused by influenza A and B viruses, with pandemics typically linked to influenza A viruses^3^. Influenza A viruses have been isolated from wild birds and belong to the genus Alphainfluenzavirus within the Orthomyxoviridae virus family^4^.

These viruses can be further classified based on the hemagglutinin (H) and neuraminidase (N) surface proteins, resulting in various subtypes [e.g. Hemagglutinin 1 and Neuraminidase 1 (H1N1), Hemagglutinin 1 and Neuraminidase 2 (H1N2), and Hemagglutinin 3 and Neuraminidase (H3N2) H1N1, H1N2, H3N2]. Some of these subtypes have caused significant pandemics in the past, such as the H1N1 “Spanish flu” in 1918 and the 2009 swine flu pandemic^4^. While H1N2 is endemic in pigs and has rarely caused human disease, other subtypes like H5N1 and H7N9 have posed significant pandemic threats^5^.

## Global implications

Human infections caused by novel influenza A subtypes can potentially lead to pandemics if the virus demonstrates efficient human-to-human transmission is distinct from currently circulating seasonal human influenza viruses, and the population has little or no immunity against it^6^. While most Influenza A(H1N2)v cases result in mild clinical illness, vigilance is essential as these viruses continue to be detected in swine populations^7^.

Exposure to pigs or pig products has been identified as a common risk factor for transmission. Though human-to-human transmission is limited, monitoring and control measures should be implemented, especially in cases of severe illness. The risk of international spread remains low as these viruses have not acquired the ability to sustain human-to-human transmission^8^.

## Vaccination and prevention

Vaccination against Influenza A(H1N2)v in humans is currently without a licensed vaccine^9^. However, ensuring the annual influenza vaccination is a crucial preventive measure^10^. While it may not target Influenza A(H1N2)v, the vaccination protects against other influenza strains, which can help reduce the overall burden of influenza-related illnesses. In addition to vaccination, preventive measures, such as avoiding contact with sick animals and contaminated substances in areas where pigs are present, are important. Regular and thorough hand hygiene, through practices such as handwashing with soap and water, is also a documented measure to reduce the risk of infection^11^. In cases where proximity to pigs is necessary, especially for individuals at a higher risk of exposure, the use of masks is advisable^11^. These measures are instrumental in limiting the transmission of Influenza A(H1N2)v.

When individuals need to interact with pigs known to be infected or potentially infected, employing personal protective equipment (PPE), such as protective clothing, gloves, and well-fitted masks covering the mouth and nose, is essential. This ensures individuals are adequately protected from direct contact with infected pigs. Public health measures, including travel restrictions, social distancing, and, when necessary, lockdowns, should be implemented by government and health authorities. These interventions aim to reduce transmission rates and mitigate the impact of outbreaks on public health^12^.

## International collaboration

Highlighting the importance of preparedness without inducing undue fear or panic among the public requires a delicate balance. Addressing this balance is needed to promote informed decision-making, encourage responsible behaviour, and foster a sense of collective responsibility without causing unwarranted anxiety. International collaboration is pivotal in preventing the spread of novel influenza viruses. As delineated by the International Health Regulations (IHR), surveillance is critical in detecting and responding to global disease threats^13^. Encouraging member countries to share timely and transparent data on influenza cases, genetic sequences, and factors influencing virus spread is imperative. Collaborative efforts must fortify global surveillance endeavours, specifically monitoring the prevalence and mutations of influenza viruses^14^. Given the role of swine as reservoirs for influenza viruses, sustained collaboration between human and animal health sectors remains essential^15^.

## Conclusion

The emergence of Influenza A(H1N2)v poses a notable global health concern. A comprehensive and coordinated response is needed with the recent confirmation of human infections. While cases generally result in mild illness, the potential for severe outcomes necessitates vigilance and effective preventive measures. Understanding the virology of Influenza A(H1N2)v is crucial, considering its potential to cause pandemics if it gains efficient human-to-human transmission capabilities. Although the risk of international spread is currently low, continuous monitoring and control measures are essential, especially given the ever-evolving nature of influenza viruses. Preventive measures, such as annual influenza vaccination, meticulous hygiene practices, mask usage in proximity to pigs, and personal protective equipment, play a pivotal role in mitigating the risk of infection. Even without a specific vaccine for Influenza A(H1N2)v, vaccination offers protection against other prevalent influenza strains, reducing the overall burden of influenza-related illnesses. In addition, international collaboration, guided by the IHR, is indispensable. Given the role of swine as reservoirs for influenza viruses, continued cooperation between human and animal health sectors is of utmost importance for effective surveillance and response to global disease threats.

## Ethical approval

Not applicable.

## Consent

Not applicable.

## Sources of funding

Not applicable.

## Author contribution

Conceptualization: N.A. Writing of the first draft: all authors. Writing of final draft: all authors.

## Conflicts of interest disclosure

The author declares no conflicts of interest.

## Research registration unique identifying number (UIN)

Ethical approval is not applicable to this editorial.

## Guarantor

Nicholas Aderinto.

## Data availability statement

No new datasets were generated for this editorial.

## Provenance and peer review

Not commissioned, externally peer-reviewed.
